# 706 High Versus Low Dose Vitamin C in Burn Care

**DOI:** 10.1093/jbcr/irac012.260

**Published:** 2022-03-23

**Authors:** Seungwon Jong, Aldin Malkoc, Kerry Fine, Tam Dao, David T Wong

**Affiliations:** California University of Science and Medicine, Ontario, California; Arrowhead Regional Medical Center, Rancho Cucamonga, California; Arrowhead Regional Medical Center, Ontario, California; University of California Riverside, Riverside, California; Arrowhead Regional Medical Center, Colton, California

## Abstract

**Introduction:**

While vitamin C is a regular part of burn management, there is no consensus on the most effective dose for a reduction in mortality, fluid resuscitation requirement, and other various clinical benefits. In this study, we aim to evaluate the potential protective effects of a higher dose of intravenous vitamin C in burn patients with greater than 40% total body surface area (TBSA) as compared to the effects on low dose oral vitamin C with lower TBSA burns.

**Methods:**

A total of 54 subjects were retrospectively reviewed with burns greater than 20% TBSA from January 2018 to 2021. In our burn unit, patients with smaller burns were given 2,500 mg PO vitamin C and larger TBSA burns were given 15,000 mg IV vitamin C within 72 hours. During this period, we found 40 patients in the low dose group and 14 patients in the higher dose group. Demographics, length of stay, length on a ventilator, fluid requirements, number of procedures, days to the first infection, and mortality were compared using the Chi-square test.

**Results:**

We found that there was a significant difference in the degree of burn on admission and reassessment between the dosing groups (30% vs. 48%, p = 0.006; 32% vs. 57%, p < 0.001). Overall fluid requirements for the first three days (9 liters vs. 25 liters, p < 0.001), length of stay (13 days vs. 38 days, p = 0.011), length on a ventilator (2 days vs. 13 days, p < 0.001), and total procedures required (1 vs. 5, p = 0.014) were also significantly higher in the group given the IV dose. No significant difference in other outcomes such as days until first infection and mortality rate were found, (p=0.451 and 0.326, respectively).

**Conclusions:**

Parameters that were statistically significant were consistent with the higher burn TBSA. Despite the group with larger surface area burns to require much higher fluid requirements (25 liters vs. 9 liters in 72 hours), high dose IV vitamin C may have been protective since the outcomes of days until first infection and mortality rate had no significant difference compared to the group with the smaller TBSA burn which should have predictably better outcomes. This clinical study supports other studies that high dose vitamin C may improve outcomes from a reduction in capillary leak to mortality but an adequately powered randomized prospective approach is needed to better define the benefits as well as dosing.

